# Antimicrobial, Anthelmintic, and Antiviral Activity of Plants Traditionally Used for Treating Infectious Disease in the Similipal Biosphere Reserve, Odisha, India

**DOI:** 10.3389/fphar.2017.00658

**Published:** 2017-10-23

**Authors:** Sujogya K. Panda, Laxmipriya Padhi, Pieter Leyssen, Maoxuan Liu, Johan Neyts, Walter Luyten

**Affiliations:** ^1^Department of Zoology, North Orissa University, Baripada, India; ^2^Department of Biology, KU Leuven, Leuven, Belgium; ^3^Department of Microbiology and Immunology, Rega Institute for Medical Research, KU Leuven, Leuven, Belgium

**Keywords:** ethnopharmacology, traditional knowledge, antibacterial, antifungal, anthelmintic, antiviral, Similipal Biosphere Reserve

## Abstract

In the present study, we tested *in vitro* different parts of 35 plants used by tribals of the Similipal Biosphere Reserve (SBR, Mayurbhanj district, India) for the management of infections. From each plant, three extracts were prepared with different solvents (water, ethanol, and acetone) and tested for antimicrobial (*E. coli, S. aureus, C. albicans*); anthelmintic (*C. elegans*); and antiviral (*enterovirus 71*) bioactivity. In total, 35 plant species belonging to 21 families were recorded from tribes of the SBR and periphery. Of the 35 plants, eight plants (23%) showed broad-spectrum *in vitro* antimicrobial activity (inhibiting all three test strains), while 12 (34%) exhibited narrow spectrum activity against individual pathogens (seven as anti-staphylococcal and five as anti-candidal). Plants such as *Alangium salviifolium, Antidesma bunius, Bauhinia racemosa, Careya arborea, Caseria graveolens, Cleistanthus patulus, Colebrookea oppositifolia, Crotalaria pallida, Croton roxburghii, Holarrhena pubescens, Hypericum gaitii, Macaranga peltata, Protium serratum, Rubus ellipticus*, and *Suregada multiflora* showed strong antibacterial effects, whilst *Alstonia scholaris, Butea monosperma, C. arborea, C. pallida, Diospyros malbarica, Gmelina arborea, H. pubescens, M. peltata, P. serratum, Pterospermum acerifolium, R. ellipticus*, and *S. multiflora* demonstrated strong antifungal activity. Plants such as *A. salviifolium, A. bunius, Aporosa octandra, Barringtonia acutangula, C. graveolens, C. pallida, C. patulus, G. arborea, H. pubescens, H. gaitii, Lannea coromandelica, M. peltata, Melastoma malabathricum, Millettia extensa, Nyctanthes arbor-tristis, P. serratum, P. acerifolium, R. ellipticus, S. multiflora, Symplocos cochinchinensis, Ventilago maderaspatana*, and *Wrightia arborea* inhibit survival of *C. elegans* and could be a potential source for anthelmintic activity. Additionally, plants such as *A. bunius, C. graveolens, C. patulus, C. oppositifolia, H. gaitii, M. extensa, P. serratum, R. ellipticus*, and *V. maderaspatana* showed anti-enteroviral activity. Most of the plants, whose traditional use as anti-infective agents by the tribals was well supported, show *in vitro* inhibitory activity against an enterovirus, bacteria (*E. coil, S. aureus*), a fungus (*C. albicans*), or a nematode (*C. elegans*).

## Introduction

It has been estimated that less than 1–10% of plant species of the world have been studied chemically and pharmacologically for their potential medicinal value (Verpoorte, 2000). For tropical forests that percentage is even much lower (Gurib–Fakim, 2005). Experts estimate that 137 species (plants or animals) are lost every single day (50,000 species in a year) due to rainforest deforestation (http://www.rain-tree.com/facts.htm#.VVhhjRtS-zM). Plants species of tropical forests produce more chemical compounds for defense against pathogens and herbivores due to the high temperatures and scarcity of water during the summer, in addition to competing for space and light which force them to develop a robust means of energy and nutrient utilization along with adequate resources for secondary metabolite production (Fyhrquist, 2007). For these reasons, many tropical plant species contain secondary metabolites with potential medical utility (Wood–Sheldon et al., 1997).

The Similipal Biosphere Reserve (https://en.wikipedia.org/wiki/Simlipal_National_Park#Flora_and_fauna and Panda, 2014) is part of the UNESCO World Network of Biosphere Reserves since 2009, contains a fauna treasure trove of 1,076 plants (1,012 wild and 64 cultivated) from 168 families, including 60 species of pteridophytes, 92 species of orchids and two gymnosperms (Saxena and Brahmam, 1994-1996).

The use of medicinal plants for various ailments is a common practice among the tribal inhabitants of the SBR and is often passed orally from generation to generation. At the current stage, the younger generation of the SBR tribes shows little interest to inherit the traditional knowledge form their elders (Panda, 2014). So, a wealth of information now risks disappearing since it is often kept secret up to an old age by the initiated. Each time a traditional healer dies without passing his knowledge on to the next generation, the tribe and the world lose thousands of years of irreplaceable knowledge about medicinal plants.

Though traditional medicines have been recognized as a part of primary health care, there is a need to evaluate scientifically the crude extracts for clinical usefulness and toxicological risk. Although there are many reports available on antimicrobial properties of medicinal plants used by the tribes of Mayurbhanj, very few systematic surveys exist, and they mostly neglect the scientific validation of this traditional knowledge. Moreover, many surveys do not take full advantage of evidence for clinical efficacy in different diseases. The objective of the present paper is therefore to evaluate the antiviral, antibacterial, antifungal, and anthelmintic activities of medicinal plants used for treating infections by the tribes of the SBR (Mayurbhanj) in a systematic and consistent manner. The results can provide more scientific evidence supporting the clinical application of these plants, and can serve as a starting point for drug discovery. For each type of test, a common causative agent viz. *Escherichia coli, Staphylococcus aureus* (antibacterial-), *Candida albicans* (antifungal-), and *enterovirus* 71 (antiviral activity) was used as target organism, while for anthelmintic testing the model organism *Caenorhabditis elegans* was used.

## Materials and methods

### Chemicals and reagents

Acetone (SZBE082SV) of analytical grade was purchased from Sigma-Aldrich Co. (USA). Absolute ethanol (E/0650DF/15) was purchased from Fischer Chemicals (UK). Sterile deionized water was produced by a water purification system (Milli-Q Reagent Water System, MA, USA). Yeast extract and Bacto™ peptone were purchased from Lab M Ltd. (Lancashire, UK). Ammonium chloride, calcium chloride, cholesterol, ciprofloxacin hydrochloride, dextrose, dimethyl sulphoxide (DMSO, molecular biology grade), levamisole hydrochloride, magnesium sulfate, (±)-miconazole nitrate salt, rupintrivir, potassium dihydrogen phosphate, di–sodium hydrogen phosphate, sodium chloride, sodium hydroxide, and sucrose were all purchased from Sigma–Aldrich (MO, USA). MTS (3–[4,5 dimethylthiazol–2–yl]–5–[3–carboxymethyl–phenyl]–2–[4–sulphophenyl]–2H–tetrazolium, inner salt), was purchased from Promega, Leiden, The Netherlands.

### Ethnobotanical studies

#### Study design and sampling procedure

Field trips to the SBR were undertaken (2012–2013) for collecting medicinal plants. Geographic coordinates for each survey site were determined in the field with a global positioning system (GPS) receiver (Garmin Etrex, Olathe, Kansas, USA). Coordinates were recorded as latitude and longitude in decimal degrees. The ethnobotanical studies were performed according to the guidelines of Singh et al. (2013). Traditional knowledge holders (local tribal baidyas (https://en.wikipedia.org/wiki/Baidya) and elders) were included in the design of the study protocol and throughout its implementation. Most of these tribal baidyas are certified and government-recognized traditional knowledge holders (TKH) and belong to Aswin Kumar Vaidya Sangha, in the Mayurbhanj district, Odisha. A semi-structured questionnaire survey provided by SRISTI (Society for Research and Initiatives for Sustainable Technologies and Institutions; http://www.sristi.org/cms/) was employed to document the traditional knowledge. Data were collected in face-to-face interviews with translation where necessary. In total 14 TKH were interviewed. All participants were male with average age of 45. No minors were approached in the present survey. Personal interviews and group discussions with the TKH revealed specific information about the plants, which was further authenticated by crosschecking. Regarding common diseases, the respondents provided information about the frequent occurrence of infection (abscess, burn, wound, skin, eye and ear, sinusitis, diarrhea, dysentery), parasitic disease (different intestinal worms, malaria), bone fracture, gout, fever, jaundice, and snakebite.

#### Ethical issues

To carry out research activities inside the Similipal Tiger Reserve (national park), permission was obtained from the Principal Chief Conservator of Forests (Odisha) and Field Director, Similipal Tiger Reserve–cum–Regional Chief Conservator of Forests, Baripada, Odisha. However, no permission was required for research activities in the villages near the transition zone of the SBR (http://www.moef.nic.in/division/biosphere-reserves). Ethnopharmacological studies of the type that we conducted fall under the authority of the Indian National Biodiversity Authority, an Autonomous and Statutory Body of the Ministry of Environment, Forest, and Climate Change, Government of India (http://nbaindia.org/link/304/1/1/home.html), and are governed by the Biological Diversity Act, 2002 (http://nbaindia.org/content/25/19/1/act.html).

Participating TKHs were contacted, and the purpose of the research project was explained to them before they gave written informed consent. Each participant of the study agreed to participate voluntarily. Participants were allowed to discontinue the interview at any time. The informed consent form that we used was developed by the National Innovation Foundation and Honey Bee Network and was obtained from the SRISTI website (http://www.sristi.org/cms/). After acceptance of the manuscript for publication, the data will be contributed to the Traditional Knowledge Digital Library (TKDL) of India (http://www.tkdl.res.in/tkdl/langdefault/common/Abouttkdl.asp?GL=Eng).

#### Botanical identification

**Figure 1:**
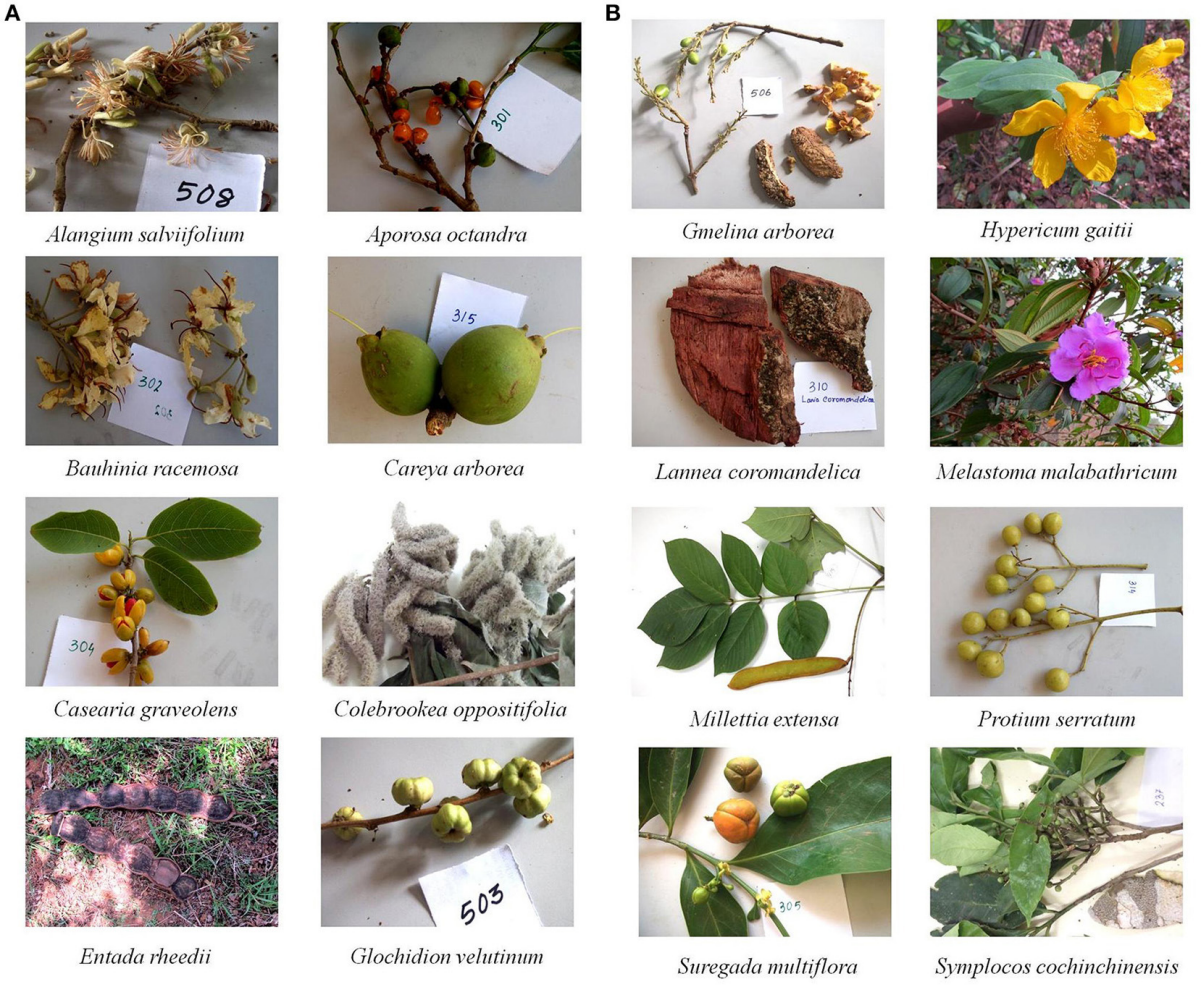
Photographs of selected plants from Similipal Biosphere Reserve.

In the field work for each plant species, the following data were noted: local name, place, collection method, parts used, preparation methods, and medicinal value. The collected plant species were identified by professional taxonomists of North Orissa University (see acknowledgments) with the help of floras (Saxena and Brahmam, 1994-1996). None of them are endangered or protected species. Identification and voucher specimen deposition of these medicinal plants was done at the Department of Botany, North Orissa University, Baripada, and pictures of selected plants are shown in Figure 1. The information on these plants with their official taxonomic name (according to the plant list- http://www.theplantlist.org), local name, medicinal use, and used part(s), GPS coordinates, origin, and voucher numbers are shown in Table 1.

**Table 1:** Ethnomedicinal uses of medicinal plants of SBR and Mayurbhanj district, India.

**Voucher No**.	**Taxonomic name**	**Local name**	**Family**	**Parts used**	**GPS coordinates and collection province**	**Medicinal uses**
NOU508/2013	*Alangium salviifolium* (L.f.) Wangerin	Baghanakhia/Ankula	Cornaceae	Leaf	21°53′30.9″ N, 86°23′55.0″ E; Nawana	Wound, infection due to snakebite
NOU100/2012	*Alstonia scholaris* (L.) R. Br.	Chatiana	Apocyanaceae	Leaf	21°54′51.4″ N, 86°27′26.1″ E; Lulung	Mouth infection, jaundice
NOU105/2012	*Anogeissus latifolia* (Roxb. ex DC.) Wall. ex Guill. & Perr.	Dha/Dhaura	Combretaceae	Bark	21°54′51.4″ N, 86°27′26.1″ E; Lulung	Diarrhea
NOU312/2013	*Antidesma bunius* (L.) Spreng.	Mutta	Phyllanthaceae	Bark, fruit	21°55′56.1″ N, 86°27′20.0″ E; Machhakandana	Infection (any), skin, diarrhea
NOU301/2013	*Aporosa octandra* (Buch.-Ham. ex D. Don) Vickery	Makania	Phyllanthaceae	Bark, leaf	21°53′30.9″ N, 86°23′55.0″ E; Nawana	Abscess, infection due to bone fracture
NOU213/2012	*Barringtonia acutangula* (L.) Gaertn.	Hinjala/Banasadina	Lecythidaceae	Leaf	21°56′51.2″ N, 86°33′19.3″ E; Sitakund	Worm infection, skin infection
NOU302/2013	*Bauhinia racemosa* Lam.	Anmata	Fabaceae	Leaf	21°46′22.5″ N, 86°31′00.6″ E; Debkund	Infection (any), skin, diarrhea
NOU207/2012	*Buchanania lanzan* Spreng.	Chara	Anacardiaceae	Bark	21°56′51.2″ N, 86°33′19.3″ E; Sitakund	Diarrhea
NOU290/2012	*Butea monosperma* (Lam.) Taub.	Palasa	Leguminosae	Flower	21°58′32.2″ N, 86°36′35.2″ E; Baldiha	Diarrhea, dysentery
NOU315/2013	*Careya arborea* Roxb.	Kumbhi	Lecythidaceae	Leaf	21°46′22.5″ N, 86°31′00.6″ E; Debkund	Skin infection
NOU315/2013	*Careya arborea* Roxb.	Kumbhi	Lecythidaceae	Bark	21°58′04.3″ N, 86°32′48.1″ E; Champagad	Diarrhea, dysentery
NOU304/2013	*Casearia graveolens* Dalzell	Girchi	Salicaceae	Leaf	21°57′27.9″ N, 86°35′26.4″ E; Lulung	Piscicide, skin infection
NOU301/2013	*Cleistanthus patulus* (Roxb.) Müll. Arg.	–	Phyllanthaceae	Leaf	21°46′22.5″ N, 86°31′00.6″ E; Debkund	Infection (any), skin, diarrhea
NOU244/2012	*Colebrookea oppositifolia* Sm.	Marang	Lamiaceae	Leaf	21°53′30.9″ N, 86°23′55.0″ E; Nawana	Sinusitis, malaria, intestinal problems
NOU215/2012	*Crotalaria pallida* Aiton	–	Fabaceae	Fruit	21°46′22.5″ N, 86°31′00.6″ E; Debkund	Intoxication, antiparasitic
NOU068/2012	*Croton roxburghii* N.P. Balakr.	Putuli	Euphorbiaceae	Leaf	21°46′22.5″ N, 86°31′00.6″ E; Debkund	Diarrhea
NOU062/2012	*Dillenia pentagyna* Roxb.	Rai	Dilleniaceae	Leaf	21°56′51.2″ N, 86°33′19.3″ E; Sitakund	Diarrhea, dysentery
NOU057/2012	*Diospyros malbarica* (Desr.) Kostel.	Kalikendu	Ebenaceae	Leaf	21°56′51.2″ N, 86°33′19.3″ E; Sitakund	Skin infection, diarrhea
NOU254/2012	*Enteda rheedii* Spreng.	–	Leguminosae	Seed	21°53′30.9″ N, 86°23′55.0″ E; Nawana	Eye infection
NOU503/2013	*Glochidion velutinum* Wight	–	Euphorbiaceae	Leaf	21°53′30.9″ N, 86°23′55.0″ E; Nawana	Infection due to bone fracture, fever
NOU506/2013	*Gmelina arborea* Roxb.	Gambhari	Lamiaceae	Leaf	21°58′04.3″N, 86°32′48.1″ E; Champagad	Infection (any), skin, malaria
NOU059/2012	*Holarrhena pubescens* (Buch.-Ham.) Wall. ex G. Don	Kuruchi	Apocynaceae	Bark	21°54′51.4″ N, 86°27′26.1″ E; Lulung	Malaria, diarrhea, dysentery
NOU059/2012	*Holarrhena pubescens* (Buch.-Ham.) Wall. ex G. Don	Kuruchi	Apocynaceae	Leaf	21°54′51.4″ N, 86°27′26.1″ E; Lulung	Skin infection, jaundice
NOU228/2012	*Hypericum gaitii* Haines	–	Hypericaceae	Leaf	21°47′27.2″ N, 86°24′02.35″ E; Nawana	Skin infection, sore throat, fever
NOU310/2013	*Lannea coromandelica* (Houtt.) Merr.	Jia	Anacardiaceae	Bark	21°56′51.2″ N, 86°33′19.3″ E; Sitakund	Worm infection, skin infection
NOU301/2013	*Macaranga peltata* (Roxb.) Müll. Arg.	Manda	Euphorbiaceae	Leaf	21°55′56.1″ N, 86°27′20.0″ E; Machhakandana	Skin infection
NOU232/2012	*Melastoma malabathricum* L.	Korali	Melastomataceae	Leaf	21°53′30.9″ N, 86°23′55.0″ E; Nawana	Infection (any), skin, diarrhea
NOU414/2013	*Millettia extensa* (Benth.) Baker	Gora	Leguminosae	Leaf	21°47′27.2″ N, 86°24′02.35″ E; Nawana	Skin infection, cough
NOU208/2012	*Mimosa rubicaulis* Lam.	Chur	Leguminosae	Leaf	21°57′27.9″ N, 86°35′26.4″ E; Lulung	Burns, wound infections (any)
NOU542/2013	*Nyctanthes arbor-tristis* L.	Haragoura/Gangasiuli	Oleaceae	Leaf	21°58′32.2″ N, 86°36′35.2″ E; Baldiha	Infection (any), skin, diarrhea, malaria, jaundice
NOU314/2013	*Protium serratum* (Wall. ex Colebr.) Engl.	Rajmoi	Burseraceae	Fruit	21°53′30.9″ N, 86°23′55.0″ E; Nawana	Tuberculosis, cough
NOU103/2012	*Pterospermum acerifolium* (L.) Willd.	Muchkunda	Sterculiaceae	Bark	21°58′04.3″ N, 86°32′48.1″ E; Champagad	Diarrhea, dysentery
NOU103/2012	*Pterospermum acerifolium* (L.) Willd.	Muchkunda	Sterculiaceae	Flower	21°58′04.3″ N, 86°32′48.1″ E; Champagad	Diarrhea
NOU103/2012	*Rubus ellipticus* Sam.	Machaokodi	Rosaceae	Leaf	21°47′27.2″ N, 86°24′02.35″ E; Nawana	Diarrhea, malaria
NOU305/2013	*Suregada multiflora* (A. Juss.) Baill.	Khakra	Euphorbiaceae	Leaf	21°46′22.5″ N, 86°31′00.6″ E; Debkund	Skin
NOU305/2013	*Suregada multiflora* (A. Juss.) Baill.	Khakra	Euphorbiaceae	Bark, seed	21°46′22.5″ N, 86°31′00.6″ E; Debkund	Diarrhea, dysentery
NOU103/2012	*Symplocos cochinchinensis* (Lour.) S.	–	Symplococeae	Leaf	21°53′30.9″ N, 86°23′55.0″ E; Nawana	Skin
NOU426/2013	*Ventilago maderaspatana* Gaertn.	Raktapichula	Rhamnaceae	Bark, leaf	21°53′30.9″ N, 86°23′55.0″ E; Nawana	Gout, bone fracture, ear, eye infection
NOU306/2013	*Wrightia arborea* (Dennst.) Mabb.	Pita karua	Apocynaceae	Leaf	21°46′22.5″ N, 86°31′00.6″ E; Debkund	Diarrhea, skin infection, asthma

### Preparation of the extract

Bark, flowers, fruit, leaves, or roots of plants were collected separately during field trips to different places of the SBR. After collection, the healthy leaves were dried at ambient temperature in the absence of sunlight for 7 days to maintain their green color and volatile oils, if present. Other parts such as bark, root, flower, fruits, were dried in an oven (Labotech Solutions, India) at 50°C for 4 days. All the materials were completely dried to prevent growth of fungi, molds, bacteria, or other microorganisms (Panda et al., 2016). The dried raw botanical material was ground to a fine powder and stored in PearlPet plastic jars, 600 gm. One gram of powder was transferred into each of three 15 mL sterile polypropylene tubes with screwcaps, and 10 mL of sterile water, absolute ethanol, or acetone were added, respectively. Extraction was performed at ambient temperature with the aid of repeated vortexing and sonication (4 × 15 min over a 24 h period) in a sonicator water bath (Branson, USA). After 1 day, the tubes were centrifuged for 10 min at 2000 RCF (Hettich Rotanta 46R, C4810, Germany) and the supernatant transferred in 1 mL aliquots to 1.5 mL Eppendorf tubes. After evaporation of the solvent in a Savant SpeedVac Concentrator (SVC 200H, Stratech Scientific, London, UK), the dried residue of 1 mL extract was re-dissolved in 200 μL water (for the aqueous extract) or 200 μL DMSO for the ethanol and acetone extracts. For the antiviral assay, 1 mL of each extract was transferred to a special tube (2D barcoded 2 mL storage tube, Thermo Scientific^*^ Abgene) and the solvent was evaporated as described above. The dry residue was dissolved in DMSO to a final stock concentration of 10 mg/mL. The samples were stored at 4°C until further testing.

### Antimicrobial test

#### Microbial strains

Bacterial strains *E. coli* (DH10B), *S. aureus* ATCC 65385 (Rosenbach), and fungal strain *C. albicans* (SC5314) (American Type Culture Collection, Manassas, Virginia, USA) (Fonzi and Irwin, 1993) were used for the antibacterial and antifungal test, respectively. For bacteria, colonies were inoculated on Mueller-Hinton (MH) agar plates, while for *Candida*, colonies were inoculated on YPD agar, and after overnight growth both plate types were sealed with parafilm and stored in a cold room (4°C).

#### Preparation of pre-culture

A single colony of *C. albicans, E. coli*, or *S. aureus* was inoculated from an agar plate in 5 mL of YPD medium (1% yeast extract, 2% peptone, and 2% dextrose) for *Candida*, or MH medium (0.2% beef extract, 1.75% casamino acids, and 0.015% soluble starch) for bacteria, in separate reaction tubes under aseptic conditions. The reaction tubes were incubated overnight while shaking at 200 rpm at 37°C.

#### Antibacterial activity test (microdilution broth) protocol

Ten μL of the test sample was transferred into the wells of a 96-well plate, as well as the positive control (ciprofloxacin, stock 100 μg/mL) and blank (solvent) controls (DMSO and water). Each well of a microdilution plate was than inoculated with 190 μL of the diluted standardized inoculum (OD = 0.003 at 620 nm). Control wells were prepared with 190 μL MH broth and 10 μL extract to correct of any absorption due to extract components. The microdilution plates were placed in a shaker-incubator at 37°C for 18 h, and then read on a Mithras LB 940 Multimode Microplate Reader (Berthold Technologies GmbH & Co. KG, Bad Wildbad, Germany) at 620 nm with a lamp energy of 13,000 using the MikroWin 2000 software package. The OD was measured at a wavelength of 620 nm and wells with a plant extract were corrected for the absorption contributed by the extract. Tests were typically carried out in duplicate. The relative inhibition (%) of the test sample was calculated by dividing the OD value of the test sample minus that of the non-inoculated extract control by the average OD of the solvent control, and multiplying by 100.

#### Antifungal test

Antifungal activity was tested against *C. albicans* in a similar way as for bacteria. Instead of MH broth, YPD broth was used with a smaller quantity of plant extract (to keep the final DMSO concentration below 2%). For antifungal activity, each well of a microdilution plate was inoculated with 196 μL of the diluted yeast suspension and 4 μL of the test solution was added. Control wells were prepared with 196 μL YPD broth and 4 μL extract to correct for any absorption due to extract components, or 4 μL of DMSO or MilliQ (solvent control), or the antifungal miconazole (200 μg/mL) in DMSO (positive control).

#### Determination of minimum inhibitory concentration (MIC_50_)

The MIC_50_ was determined using the 96–well microdilution method as described earlier in section Antibacterial Activity Test (Microdilution Broth) Protocol. The dry weight of each extract was determined on an analytical balance (Sartorius ME235P, Germany). The dry residue was dissolved in 200 μL DMSO (sterile water for aqueous extracts), and a 2–fold serial dilution (up to 32–fold) was prepared in a 96–well conical bottom (V) microplate. Data from dose-response experiments were represented as the percentage of inhibition, and analyzed with Prism™ (GraphPad Prism 5.0 Software Inc., San Diego, CA). The MIC_50_ for each growth condition was calculated by fitting the data to a non-linear least-squares sigmoid regression curve.

### Anthelmintic activity

#### Culture, maintenance, and synchronization of *Caenorhabditis elegans (C. elegans)* strains

The N_2_ wild-type *C. elegans* strain was grown on Nematode Growth Medium (NGM) in Petri dishes containing a lawn of the bacterium *E. coli* OP50 (Brenner, 1974). Synchronized populations were obtained by a modified alkaline bleaching method (Lenaerts et al., 2008). Briefly, worm culture plates with eggs and egg-laying adults were washed with M9 buffer and incubated with freshly prepared bleaching solution, followed by three washes with M9. Then, 3.5 mL of worm suspension was added to 1.5 mL of alkaline hypochlorite solution (1 mL of bleach and 0.5 mL of 5 M NaOH). The suspension was shaken for 5 min, and 5.0 mL of a sucrose solution (60% w/v) was added. Then, 1 mL of M9 buffer was gently added to the mixture, followed by centrifugation at 290 RCF (Hettich Rotanta 46R, C4810, Germany) for 3 min. After centrifugation, the eggs were collected and washed five times with M9 buffer were. Eggs in M9 were kept on a rotator at 20°C overnight in the absence of light to hatch into L1 larvae (L1s). After 24 h of incubation, L1s were transferred onto a fresh NGM plate seeded with an *E. coli* OP50 lawn and grown at 20°C (L2s). “L4” larvae were isolated from those plates after 48 h were washed four times with M9 buffer before being used for assays with plant extracts.

#### Anthelmintic assay

The anthelmintic assay was carried out in a 96-well microplate (flat-bottom, TPP Techno Plastic Products AG, Switzerland). Synchronized *C. elegans* (15 μL, ~45 L4 larvae) were added to each well of a 96-well microplate containing 184 μL of *E. coli* OP50 culture (OD = 0.5 at 620 nm). Subsequently, 1 μL of plant extract was added to each well; DMSO (1 μL) was used in a separate well as a solvent control. After mixing, the 96-well microplate was placed into a WMicroTracker (Phylumtech, Argentina) apparatus and incubated for 16 h at 20°C. The movement of worms in each well was measured every 30 min and recorded by the WMicroTracker. The percentage of the average movement over 16 h of test samples with extract compared to the DMSO control, was used to estimate the relative anthelmintic activity. Levamisole (final concentration 50 μM) was systematically used as a positive control.

### Antiviral test

The EV71 BrCr laboratory-adapted strain was used at a low multiplicity of infection (MOI) in a standardized antiviral assay as described earlier (Martínez-Gualda et al., 2017). Briefly, a serial dilution of the extract(s) was prepared in assay medium that was added to an empty microtiter 96-well plate, after which the virus inoculum was added, followed by a suspension of freshly harvested rhabdosarcoma (RD) cells (2 × 10^4^ cells/well). While the cells were settling to the bottom, the virus was allowed to infect them in the presence of extract. The assay plates were incubated at 37°C, 5% CO_2_ with virus inoculum and compounds until full virus-induced cell death was observed in the untreated, infected controls (3–4 days post-infection). Subsequently, the antiviral effect was quantified using a colorimetric readout with 3- (4,5-dimethylthiazol-2-yl)-5-(3-carboxymethoxyphenyl)-2-(4-sulfophenyl)-2H-tetrazolium/phenazine methosulfate (MTS/PMS method) and the concentration of compound at which 50% inhibition of virus-induced cell death would be observed (EC_50_) was calculated from the antiviral dose-response curves. A similar assay setup was used to determine the adverse effect of an extract or test compound on uninfected, treated cells for calculation of the CC_50_ (concentration of sample that reduces overall cell health by 50% as determined by the MTS/PMS method). The final, maximal DMSO concentration that was reached in the assay wells with the highest sample input (1%) was well-tolerated by the cells. Rupintrivir was used as a positive control (Martínez-Gualda et al., 2017). In addition, the selectivity index (SI) was calculated as the ratio of the CC_50_ for cell growth to the EC_50._The Selectivity Surface-SS, was determined from plots (CPE and CC curve) and the Therapeutic Index-TI was calculated as (SS^*^10logSI) (Supplementary Material).

## Results

### Ethnomedicinal documentation

**Figure 2:**
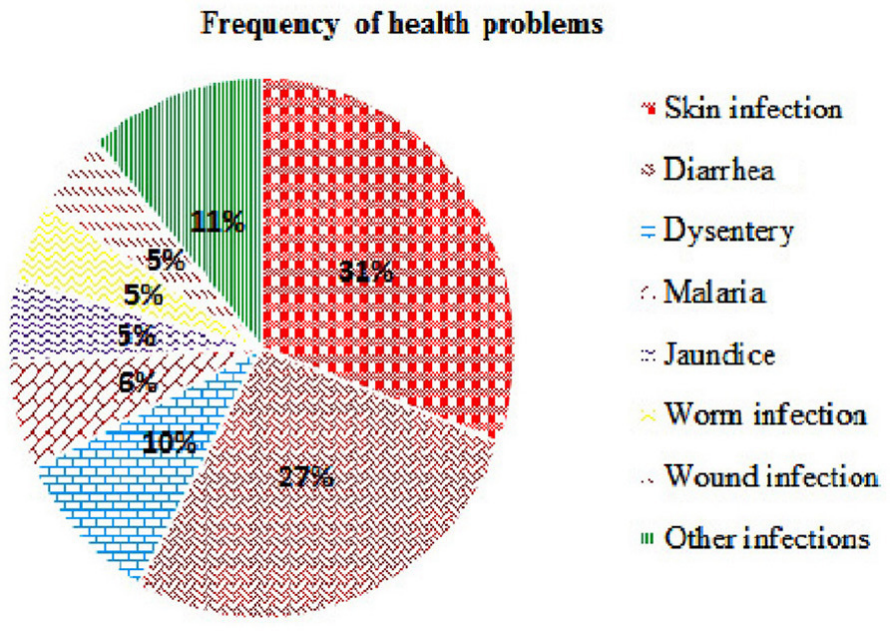
Frequency of health problems attributed by the tribals of SBR.

**Figure 3:**
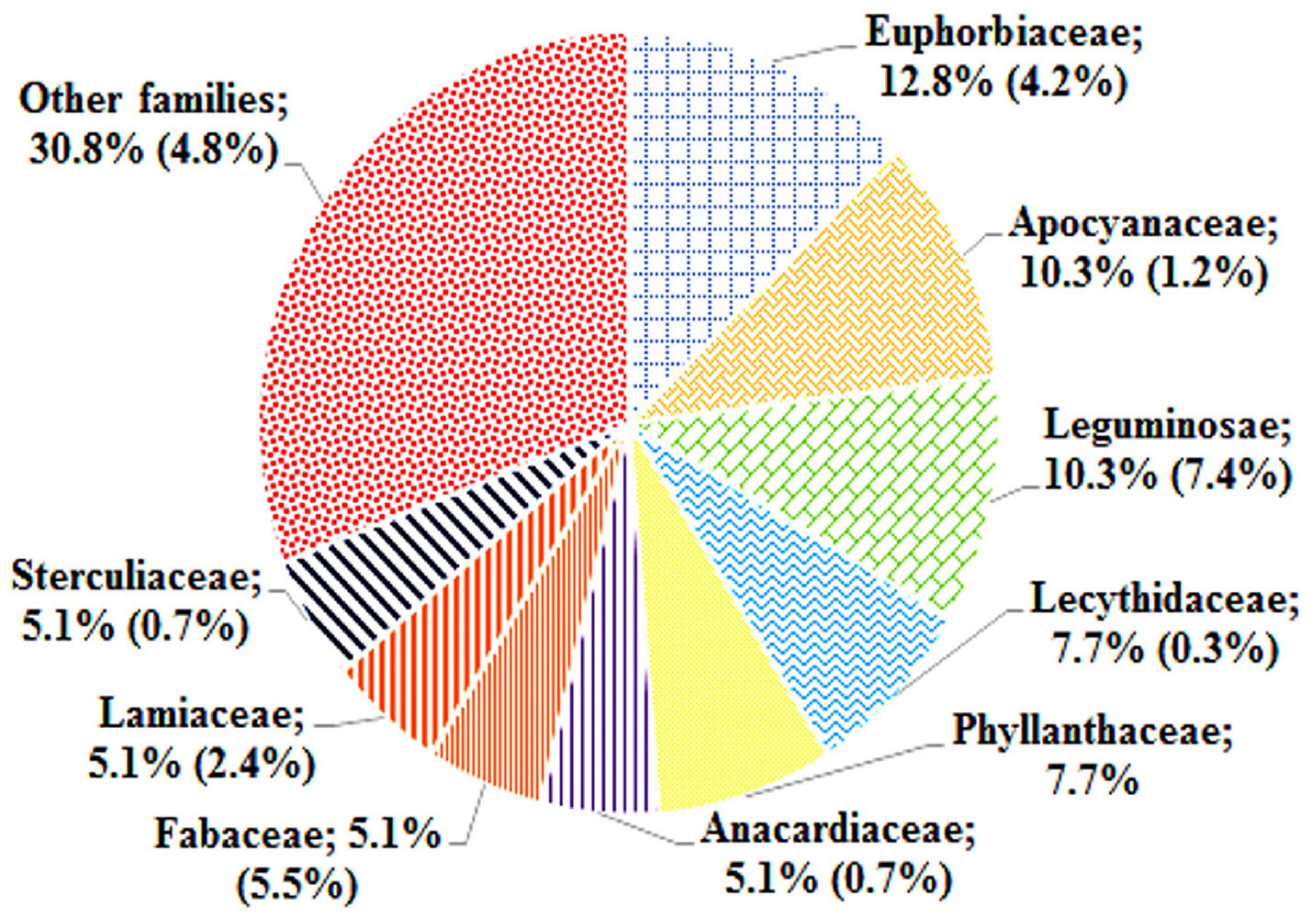
Distribution of plant families frequently used by the tribals of SBR compared to frequency of same families found in the broad survey of SBR plants (Girach et al., 1999).

The ethnomedicinal uses of 35 plant species belonging to 21 families were recorded from tribes of the SBR and periphery (Table 1). The information on these plants with botanical names (arranged alphabetically), voucher number, local name, taxonomic family, used plant part, origin, and medicinal uses is shown in Table 1. Most of the reported medicinal uses pertained to the treatment of infections such as skin infection (19), diarrhea (17), dysentery (defined as bloody diarrhea) (6), malaria (4), jaundice (3), parasitic (3), wound (3) or other infections of e.g., eye, ear, mouth, or throat, or for bone fractures, snake bites etc. (7) (Figure 2). According to the number of given uses, the most represented families are Euphorbiaceae (5), Apocynaceae (4), Leguminosae (4), Lecythidaceae (3), and Phyllanthaceae (3) (Figure 3).

### Antimicrobial activity

**Figure 4:**
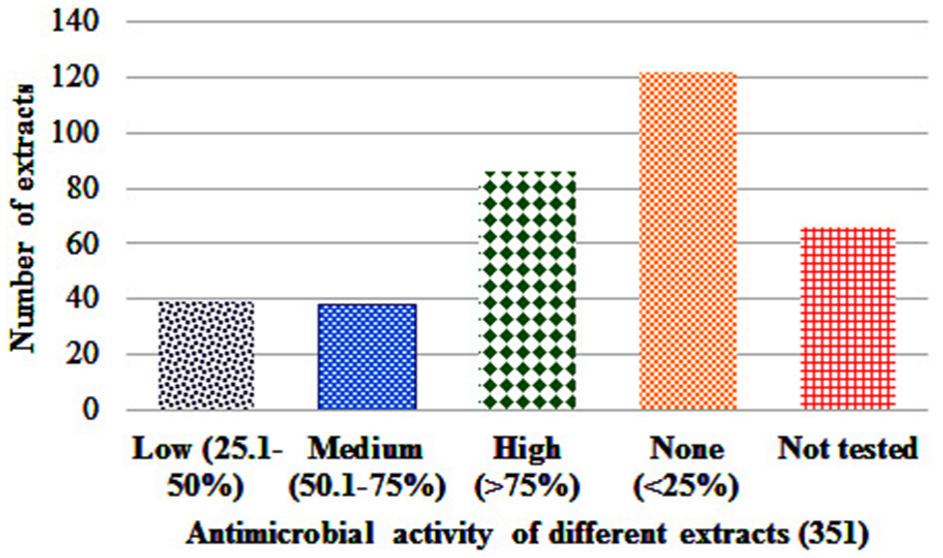
Antimicrobial activity score of different extracts.

**Figure 5:**
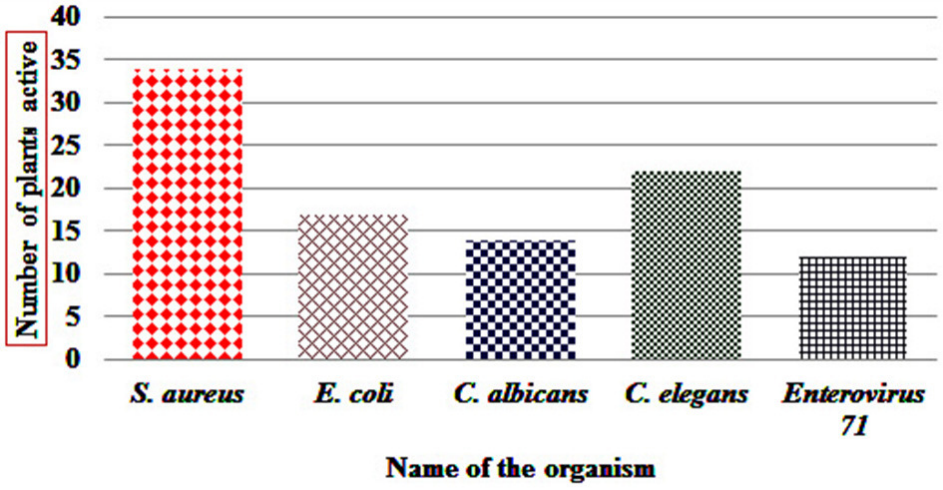
Number of SBR plants with at least one extract showing activity (>50%) against tested organism.

Antibacterial activity in microdilution broth assays with crude extracts (aqueous, acetone, and ethanol) of different plant parts of different species is presented in Table 2 (percentages of inhibition surpassing 50 are underlined). In total, 285 extracts were tested, of which 86 extract showed high activity while 122 extracts did not show activity (Figure 3). Additionally, 38 extracts had medium—while 39 extracts had low antimicrobial activity (Figure 3). Most activity was detected in ethanol extracts, followed by acetone and aqueous extracts. Of the 35 plants screened, 17 showed activities in at least one extract against *E. coli*, 34 against *S. aureus*, and 14 against *C. albicans* (Figures 4, 5). Among the tested plants, six showed activity against *E. coli* in the acetone extracts, 10 in the ethanol extracts, and 11 in water extracts. In a few cases, negative values were calculated for the % inhibition. Taken at face value, this could mean that the extract enhanced (rather than inhibited) bacterial growth. This cannot be excluded, but more likely the negative values are due to a combination of experimental variation and an overcorrection for colored compounds in the control samples without bacteria. This may occur when bacteria metabolize some of the colored components in the extract.

**Table 2:** Antimicrobial activity of selected extracts of plants collected from SBR, India (growth inhibition in % compared to the solvent, OD at 620 nm).

**Plant name**	**Parts used**	***E. coli***			***S. aureus***			***C. albicans***		
		**Acetone**	**Water**	**Ethanol**	**Acetone**	**Water**	**Ethanol**	**Acetone**	**Water**	**Ethanol**
*A. salviifolium*	Leaf	8	95	8	55	96	60	6	–16	5
*A. scholaris*	Leaf	27	45	48	98	80	97	26	6	62
*A. latifolia*	Bark	–	–	42	–	–	73	–	–	46
*A. bunius*	Bark	14	–19	11	54	97	84	15	26	18
*A. octandra*	Leaf	77	47	30	–21	99	71	13	18	–24
*B. acutangula*	Leaf	–	–	33	–	–	85	–	–	–2
*B. racemosa*	Leaf	11	84	13	66	65	64	–2	11	3
*B. lanzan*	Bark	–	–	47	–	–	78	–	–	24
*B. monosperma*	Flower	–	–	43	–	–	74	–	–	54
*C. arborea*	Leaf	–	–	63	–	–	99	–	–	56
*C. arborea*	Bark	18	–20	13	96	92	95	89	34	90
*C. graveolens*	Leaf	24	78	47	59	63	38	4	50	11
*C. patulus*	Leaf	11	–2	7	88	100	48	23	21	12
*C. oppositifolia*	Leaf	24	–5	22	98	87	100	33	114	37
*C. pallida*	Fruit	12	95	100	77	46	20	1	78	3
*C. roxburghii*	Leaf	–	–	89	–	–	75	–	–	52
*D. pentagyna*	Leaf	–	–	68	–	–	83	–	–	25
*D. malbarica*	Leaf	–	–	25	–	–	79	–	–	82
*E. rheedii*	Seed	13	47	13	18	34	24	10	7	1
*G. velutinum*	Leaf	10	–8	18	82	63	–21	10	12	26
*G. arborea*	Leaf	18	3	8	94	21	–19	16	13	74
*H. pubescens*	Bark	90	82	98	97	–3	100	9	47	70
*H. pubescens*	Leaf	100	66	96	–1	–4	98	17	32	27
*H. gaitii*	Leaf	18	15	20	99	100	85	20	18	25
*L. coromandelica*	Bark	–	–	95	–	–	99	–	–	39
*M. peltata*	Leaf	71	36	77	118	64	112	53	35	86
*M. malabathricum*	Leaf	100	72	8	93	–3	97	93	17	110
*M. extensa*	Leaf	26	11	24	93	–22	75	21	78	14
*M. rubicaulis*	Leaf	8	78	10	76	74	55	6	44	27
*N. arbortristis*	Leaf	8	–20	–3	93	–2	–14	6	6	5
*P. serratum*	Fruit	–1	–33	0	100	83	62	90	33	90
*P. acerifolium*	Bark	–	–	18	–	–	48	–	–	26
*P. acerifolium*	Flower	–	–	88	–	–	93	–	–	66
*R. ellipticus*	Leaf	7	86	10	99	95	100	100	59	88
*S. multiflora*	Leaf	13	77	13	105	85	–6	89	11	75
*S. multiflora*	Bark	20	86	22	103	71	–7	81	–27	71
*S. cochinchinensis*	Leaf	9	–17	17	47	58	18	–14	5	23
*V. maderaspatana*	Leaf	35	14	24	106	11	98	71	12	22
*W. arborea*	Leaf	91	88	62	94	99	25	12	39	20
Solvent control	DMSO	0	–	0	0	–	0	0	–	0
	Water	–	0	–	–	0	–	–	0	–
Antibiotic–ciprofloxacin		97	98	97	96	105	104	–	–	–
Antifungal–miconazole		–	–	–	–	–	–	94	91	92

*(–) Data absent, % growth inhibition data were rounded to the nearest integer. Underlined values represent above 50 inhibition*.

Plants that showed more than 75% inhibition with the broth dilution method were further tested by 2-fold serial dilution to evaluate their MIC. The MIC_50_ of the selected extracts against *E. coli, S. aureus*, and *C. albicans* are summarized in Table 3. Most of the test extracts inhibited growth at less than 1,000 μg/mL. The MIC_50_ value range from (100–1,400 μg/mL), (50–1,370 μg/mL), and (150–780 μg/mL) against *E. coli, S. aureus*, and *C. albicans*, respectively.

**Table 3:** Determination of MIC_50_ of selected extracts (concentration in μg/mL).

**Plant name**	**Parts used**	***E. coli***			***S. aureus***			***C. albicans***		
		**Acetone**	**Water**	**Ethanol**	**Acetone**	**Water**	**Ethanol**	**Acetone**	**Water**	**Ethanol**
*A. salviifolium*	Leaf	–	580	–	–	750	–	–	–	–
*A. scholaris*	Leaf	–	–	–	1,170	1,370	870	–	–	–
*A. latifolia*	Bark	–	–	–	–	–	1,130	–	–	–
*A. bunius*	Bark	–	–	–	–	960	100	–	–	–
*A. octandra*	Leaf	230	–	–	–	530	500	–	–	–
*B. acutangula*	Leaf	–	–	–	–	–	360	–	–	–
*B. racemosa*	Leaf	–	950	–	–	–	–	–	–	–
*B. lanzan*	Bark	–	–	–	–	–	780	–	–	–
*B. monosperma*	Flower	–	–	–	–	–	270	–	–	–
*C. arborea*	Leaf	–	–	–	–	–	200	–	–	–
*C. arborea*	Bark	–	–	–	280	800	410	410	-	560
*C. graveolens*	Leaf	–	300	–	–	–	–	–	–	-
C. patulus	Leaf	–	–	–	60	340	–	–	–	–
*C. oppositifolia*	Leaf	–	–	–	400	1,000	340	–	700	–
*C. pallida*	Fruit	–	1,030	100	50	–	–	–	190	–
*C. roxburghii*	Leaf	–	–	300	–	–	310	–	–	–
*D. pentagyna*	Leaf	–	–	–	–	–	580	–	–	–
*D. malbarica*	Leaf	–	–	–	–	–	350	–	–	720
G. velutinum	Leaf	–	–	–	150	–	-	–	–	–
G. arborea	Leaf	–	–	–	520	–	-	–	–	780
*H. pubescens*	Bark	260	990	320	100	–	610	–	–	–
*H. pubescens*	Leaf	310	710	300	–	–	770	–	–	–
H.*gaitii*	Leaf	–	–	–	480	860	410	–	–	–
*L. coromandelica*	Bark	–	–	1,000	–	–	730	–	–	–
*M. peltata*	Leaf	430	–	230	390	–	560	–	–	460
*M. malabathricum*	Leaf	130	1,050	–	680	–	600	390	–	480
*M. extensa*	Leaf	–	–	–	140	–	210	-	360	-
M. rubicaulis	Leaf	–	560	–	250	1,700	–	–	–	–
N. arbortristis	Leaf	–	–	–	1,280	-	–	–	–	–
P. serratum	Fruit	–	–	–	470	310	530	380	-	600
*P. acerifolium*	Bark	–	–	–	–	–	–	–	–	–
*P. acerifolium*	Flower	–	–	1,400	–	–	50	–	–	–
*R. ellipticus*	Leaf	–	450	–	200	400	160	240	–	220
S. multiflora	Leaf	–	650	–	150	630	–	710	–	390
S. multiflora	Bark	–	250	–	170	620	–	260	–	350
V. maderaspatana	Leaf	–	–	–	140	–	190	150	–	–
W. arborea	Leaf	170	450	–	180	170	–	–	–	–

*(–) Data absent; MIC_50_ value were rounded to the nearest integer*.

Except for *Entada rheedii*, all tested plants showed inhibitory activity against *S. aureus* at least with one extract (Table 2). There was not much difference in activity across the different extracts of the same plant when tested against *S. aureus*. Plants such as *A. salviifolium, A. scholaris, Antidesma bunius, B. racemosa, C. arborea, C. patulus, C. oppositifolia, H. gaitii, H. pubescens, M. malabathricum, R. ellipticus*, and *P. serratum* have inhibitory activity against *S. aureus* with all three extracts (acetone, water, and ethanol). Plants such as *A. salviifolium*, *A. octandra, B. acutangula, B. racemosa, C. arborea, C. graveolens, C. pallida, C. roxburghii*, *D. pentagyna, H. pubescens, L. coromandelica, M. peltata, M. malabathricum, M. rubicaulis, R. ellipticus, S. multiflora*, and *W. arborea* showed broad-spectrum activity by inhibiting both Gram-positive and Gram-negative bacteria.

Plants such as *A. scholaris, B. monosperma, C. arborea, C. graveolens, C. pallida*, C. *roxburghii, H. pubescens, D. malbarica, G. arborea, M. peltata, P. serratum, P. acerifolium, R. ellipticus*, and *S. multiflora* showed activity against *C. albicans* (Table 2). Like for *E. coli, C. albicans* also responded differently to extracts of the same plant in different solvents. Ethanol is the best overall extractant (14 plants) followed by acetone (8) and water (3). *C. arborea, C. graveolens, C. pallida, C. roxburghii*, *H. pubescens, M. peltata, R. ellipticus*, and *S. multiflora* were active against all three test strains: *E*. *coli, S. aureus*, and *C. albicans*.

### Anthelmintic activity

Table 4 shows the % inhibition (those surpassing 50 are underlined) on *C. elegans* after treatment with 1 μl of extract. Out of the 35 test plants, 22 plant viz. *A. salviifolium, A. bunius, A. octandra, B. acutangula, C. graveolens, C. pallida, C. patulus, G. arborea, H. pubescens, H. gaitii, L. coromandelica, M. peltata, M. malabathricum, M. extensa, N. arbor-tristis, P. serratum, P. acerifolium, R. ellipticus, S. multiflora, S. cochinchinensis, V. maderaspatana*, and *W. arborea* showed promising activity.

**Table 4:** Anthelmintic activity of selected extracts of plants collected from SBR, India.

**Taxonomic name**	**Parts used**	**%, relative movement compared to the solvent**		
		**Acetone**	**Water**	**Ethanol**
*A. salviifolium*	Leaf	79 ± 9	119 ± 5	77 ± 10
*A. scholaris*	Leaf	–	–	73 ± 24
*A. latifolia*	Bark	–	–	25 ± 6
*A. bunius*	Bark	24 ± 3	81 ± 10	66 ± 6
*A. octandra*	Leaf	15 ± 5	62 ± 6	16 ± 17
*B. acutangula*	Leaf	–	–	47 ± 4
*B. racemosa*	Leaf	100 ± 16	73 ± 25	89 ± 10
*B. lanzan*	Bark	–	–	99 ± 2
*B. monosperma*	Flower	–	–	96 ± 8
*C. arborea*	Leaf	–	–	72 ± 8
*C. arborea*	Bark	72 ± 10	101 ± 16	77 ± 16
*C. graveolens*	Leaf	28 ± 3	79 ± 5	83 ± 11
*C. patulus*	Leaf	45 ± 1	86 ± 6	69 ± 16
*C. oppositifolia*	Leaf	55 ± 7	99 ± 8	83 ± 2
*C. pallida*	Fruit	37 ± 6	132 ± 11	101 ± 1
*C. roxburghii*	Leaf	–	–	86 ± 1
*D. pentagyna*	Leaf	–	–	110 ± 3
*D. malbarica*	Leaf	–	–	83 ± 8
*E. rheedii*	Seed	61 ± 6	112 ± 33	88 ± 4
*G. velutinum*	Leaf	72 ± 10	86 ± 4	116 ± 8
*G. arborea*	Leaf	39 ± 5	126 ± 20	34 ± 9
*H. pubescens*	Bark	–	–	35 ± 4
*H. pubescens*	Leaf	–	–	37 ± 10
*H. gaitii*	Leaf	30 ± 9	80 ± 5	56 ± 2
*L. coromandelica*	Bark	–	–	16 ± 8
*M. peltata*	Leaf	40 ± 5	92 ± 2	53 ± 4
*M. malabathricum*	Leaf	66 ± 15	66 ± 15	31 ± 12
*M. extensa*	Leaf	43 ± 4	88 ± 3	11 ± 13
*M. rubicaulis*	Leaf	66 ± 17	100 ± 2	72 ± 8
*N. arbortristis*	Leaf	35 ± 4	79 ± 11	36 ± 8
*P. serratum*	Fruit	37 ± 11	80 ± 19	28 ± 1
*P. acerifolium*	Bark	–	–	92 ± 2
*P. acerifolium*	Flower	–	–	55 ± 2
*R. ellipticus*	Leaf	69 ± 13	104 ± 3	49 ± 1
*S. multiflora*	Leaf	96 ± 8	110 ± 6	62 ± 4
*S. multiflora*	Bark	82 ± 11	82 ± 37	36 ± 3
*S*. cochin*chinensis*	Leaf	108 ± 6	35 ± 16	79 ± 16
*V. maderaspatana*	Leaf	41 ± 9	72 ± 25	28 ± 11
*W. arborea*	Leaf	49 ± 1	108 ± 16	36 ± 18
Levamisole		48 ± 3	53 ± 4	41 ± 10

*(–) Data absent, all values are listed as mean ± SD; data were rounded to the nearest integer. Underlined values represent above 50 inhibition*.

### Antiviral activity

The effect of different plant extracts on the inhibition of enterovirus (type 71, BrCr) is presented in Table 5. Of the 35 plants tested, 5 acetone and 5 ethanol extracts, as well as 10 aqueous extracts showed potent inhibitory activity (higher than 50%). These active extracts were subsequently tested for cytotoxicity, and the effective concentration protecting 50 or 90% of the cells (EC_50_ and EC_50_) as well as cytotoxic concentration (CC_50_) are presented in Table 5. Additionally, the Selectivity Index (SI), Selectivity Surface (SS), and Therapeutic Index (TI) were calculated from the dose–response curves (Supplementary Figure 1). Among these plants, *A. bunius, C. graveolens, C. patulus, C. oppositifolia, H. gaitii, M. extensa, P. serratum, R. ellipticus*, and *V. maderaspatana* showed the best activity and could be suitable for finding antiviral compounds.

**Table 5:** Antiviral and cytotoxic activity of selected extracts of plants collected from SBR, India.

**Plant name**	**Parts used**	**Extract tested**	**EC_50_(μg/ml)**	**EC_90_(μg/ml)**	**Max. % inhibition**	**CC_50_(μg/ml)**	**SI**	**SS**	**TI**
*A. latifolia*	Bark	Acetone	66	>100	53	77	1	0	0
*A. bunius*	Bark	Ethanol	71 ± 22	87 ± 3	63 ± 6	98 ± 3	2 ± 1	1 ± 2	1 ± 1
*B. racemosa*	Leaf	Ethanol	43 ± 6	>100	79 ± 1	>75	>2	>3	>1
*C. graveolens*	Leaf	Aqueous	3 ± 1	4 ± 1	100 ± 0	>100	>28	>18	>26
*C. patulus*	Leaf	Aqueous	35 ± 15	>100	85 ± 19	>75	>2	>3	>2
*C. oppositifolia*	Leaf	Aqueous	82 ± 17	>100	63 ± 1	>100	>1	>1	>1
*G. velutinum*	Leaf	Acetone	9	19	70	26	3	4	2
*H. gaitii*	Leaf	Aqueous	70 ± 26	>100	69 ± 6	>100	>2	>3	>1
*M. peltata*	Leaf	Aqueous	20 ± 1	81 ± 4	79 ± 7	100 ± 0	5 ± 0	11 ± 3	8 ± 2
*M. malabathricum*	Leaf	Acetone	14 ± 2	>75	62 ± 4	20 ± 0	2 ± 1	1 ± 0.1	0.1 ± 0.1
*M. malabathricum*	Leaf	Ethanol	46 ± 5	>100	76 ± 1	>75	>2	>4	>1
*M. extensa*	Leaf	Aqueous	42 ± 17	>100	90 ± 14	>100	>2	>4	>2
*M. rubicaulis*	Leaf	Aqueous	54 ± 1	>100	81 ± 1	>100	>2	>4	>1
*P. serratum*	Fruit	Ethanol	15 ± 7	57 ± 44	99 ± 2	>100	>5	>12	>10
*R. ellipticus*	Leaf	Aqueous	5 ± 5	8 ± 6	88 ± 18	59 ± 3	18 ± 16	28 ± 17	36 ± 33
*R. ellipticus*	Leaf	Ethanol	13 ± 6	15 ± 0	74 ± 1	29 ± 4	3 ± 1	3 ± 2	2 ± 1
*V. maderaspatana*	Leaf	Aqueous	74 ± 44	>100	70 ± 28	>100	>1	>1	>1
*W. arborea*	Leaf	Acetone	12	–	59	18	1	1	0
*W. arborea*	Leaf	Aqueous	12 ± 5	>75	89 ± 1	>100	>10	>11	>11
Rupintrivir			0.02 ± 0.001μM	0.03 ± 0.001μM	100 ± 0	>10μM	–	–	–

*EC_50_ = 50% Effective Concentration (concentration at which 50% inhibition of virus replication is observed)*.

*EC_90_ = 90% Effective Concentration (concentration at which 90% inhibition of virus replication is observed)*.

*CC_50_ = 50% Cytostatic/Cytotoxic Concentration (concentration at which 50% adverse effect is observed on RD cells in parallel with antiviral assay)*.

*SI = Selectivity Index (CC_50_/EC_50_)*.

*SS = Selectivity Surface (integrated surface delineated by the EC_50_ curve, the CC_50_ curve and the 50% horizontal)*.

TI = Therapeutic Index (SS^*^10logSI).

*(–) Data absent; all values are listed as mean ± SD; data were rounded to the nearest integer*.

## Discussion

Since ancient time, plants are well-known as a source of medicine for the treatment of diverse diseases, and they continue to serve as the basis for many pharmaceuticals used today. They are a rich bio-resource for drugs of traditional medicinal systems, modern medicines, nutraceuticals, food supplements, folk medicines, pharmaceuticals, intermediates, and chemicals for synthetic drugs (Hammer et al., 1999). Medicinal uses are well-described in the Indian Ayurveda (Patwardhan, 2005), in Traditional Chinese Medicine (Wan et al., 2016), and in various European historical documents (Ginsburg and Deharo, 2011). However, indigenous knowledge in a particular region is an important component of traditional medicine, which is widely practiced by the tribal communities throughout India. The traditional medicines not only provide valuable clues for finding new drugs, but also help to shift the drug discovery paradigm from “finding new-entity drugs” to “combining existing agents,” and might even guide the combinations between such agents (Wagner and Ulrich–Merzenich, 2009).

From the different test performed in the present study, we conclude that *C. arborea, C. graveolens, C. pallida, C. roxburghii*, *H. pubescens, M. peltata, R. ellipticus*, and *S. multiflora* could be potential sources for broad-spectrum antibiotics as they inhibit all three test strains: *E*. *coli* (a model Gram-), *S. aureus* (a model Gram +), and *C. albicans* (the most common human pathogenic fungus). Moreover, plants such as *A. salviifolium, A. bunius, B. racemosa, C. patulus, C. oppositifolia, H. gaitii*, and *P. serratum* can be a useful source for narrow-spectrum antibiotics against *S. aureus*, while plants such *A. scholaris, B. monosperma, D. malbarica, G. arborea*, and *P. acerifolium* could yield antifungal agents. Thus, the indigenous knowledge by the tribals of the SBR appears to be a promising source for antimicrobial agents.

In this study, 19 plants parts were used traditionally to treat various skin infections, 17 plant parts for diarrhea and six plant parts for dysentery. In the present study, it was experimentally demonstrated that most plants used by tribals of Similipal (Mayurbhanj district), are potential sources of natural antibiotics. According to Williamson et al. (1996), the extraction procedure can be guided by how the plant is used in folk medicine. The tribals of SBR mostly use aqueous extracts (infusion/decoction). We observed that aqueous extracts frequently produce inhibitory effects against both bacterial strains, while they are rarely effective against *C. albicans*. We also found that some plants show activity in both polar and non-polar solvents, and sometime in all three extracts. This suggests that different active compounds are present, which may show additive or synergistic effects when the complete plant is used for treatment.

The plant extracts were most often active on *S. aureus*, followed by *E. coli* and finally *C. albicans*. Plant extracts typically show stronger inhibition on Gram-positive than Gram-negative bacteria (Desta, 1993). The latter are in general more resistant to the action of antimicrobials compared to the former because of a more complex cell wall (Silhavy et al., 2010).

Like bacterial and fungal infections, parasitic infections pose also major global health problems in humans and animals. Nematode parasites cause serious problems in the livestock industry due to drug resistance in several helminth species (Prichard, 1994; Perry and Randolph, 1999). The traditional practitioners have extensive knowledge about the treatment of parasitic infections. Therefore, we screened for extracts active on *C. elegans*, which has recently received increased attestation as a model organism for drug discovery (Desalermos et al., 2011). In the present study, of the 83 plant extracts tested, 30 had pronounced anthelmintic activity (>50% activity inhibition). More than half of the extracts show some activity (defined arbitrarily as activity inhibition by more than 25%). This suggests the accuracy of the use of plants by the tribes of the SBR. The plant extracts that inhibited the activity of *C. elegans* can be promising starting points in the search for anthelmintic drugs.

In addition to antibacterial, antifungal, and anthelmintic properties, the crude extracts were also evaluated for antiviral activity using enterovirus type 71, BrCr strain. The genus *Enterovirus* is responsible for a wide variety of diseases including meningitis, myocarditis, encephalitis and respiratory diseases (Bruu, 2002). There is currently no effective drug for the treatment of EV71 infections. The EC_50_ and CC_50_ were determined for active extracts from the dose–response curves. Considering the Selectivity Index, Selectivity Surface and Therapeutic Index, the crude extracts of *A. bunius* (bark), *C. graveolens* (leaf), *C. patulus* (leaf), *C. oppositifolia* (leaf), *H. gaitii* (leaf), *M. extensa* (leaf), *P. serratum* (fruit), *R. ellipticus* (leaf), and *V. maderaspatana* (leaf) demonstrate potential anti-enteroviral activity.

In the present study for selected extracts, cytotoxic activity was determined by calculating the % of surviving cells across a range of concentrations (1–100 μg/mL). Several plants show significant antiviral effects with no significant cytotoxicity, thus proving safe at least for mammalian cells.

Based on our review of the literature, the antimicrobial activity of *A. octandra, C. graveolens, C. patulus, D. pentagyna, G. velutinum, H. gaitii, L. coromandelica, M. extensa, M. rubicaulis, P. serratum, R. ellipticus, S. multiflora, S. cochinchinensis* and *W. arborea* has not been documented so far, while plants such as *A. salviifolium* (Jain et al., 2010), *A. scholaris* (Khan et al., 2003; Hussain et al., 2010), *A. latifolia* (Govindarajan et al., 2006; Patil and Gaikwad, 2010), *A. bunius* (Lizardo et al., 2015), *B. acutangula* (Sahoo et al., 2008), *B. racemosa* (Rashed and Butnariu, 2014), *B. lanzan* (Pattnaik et al., 2013), *B. monosperma* (Tiwari et al., 2012; Sahu and Padhy, 2013), *C. arborea* (Kumar et al., 2006) *C. oppositifolia* (Mahapatra et al., 2013), *C. pallida* (Ukil et al., 2016), *C. roxburghii* (Thatoi et al., 2008; Panda et al., 2010a,b), *D. malbarica* (Panda et al., 2012), *G. arborea* (Khan et al., 2003; El–Mahmood et al., 2010), *H. pubescens* (Chakraborty and Brantner, 1999; Siddiqui et al., 2012), *M. malabathricum* (Alwash et al., 2014), *M. peltata* (Bijesh and Sebastian, 2013), *N. arbor-tristis* (Aggarwal and Goyal, 2013), *P. acerifolium* (Panda and Dutta, 2012), and *V. maderaspatana* (Kawde et al., 2014) were reported before to show antimicrobial effects. Likewise, *A. salviifolium* (Pandey, 2012), *B. acutangula* (Padmavathi et al., 2011), *C. pallida* (Panda et al., 2014), *G. arborea* (Panda et al., 2015), *H. pubescens* (Satpute et al., 2014), *M. malabathricum* (Suteky and Dwatmadji, 2011), and *N. arbor-tristis* (Shruti et al., 2009) were reported earlier for anthelmintic properties, while plants such as *A. bunius, A. octandra, C. graveolens, C. patulus, H. gaitii, L. coromandelica, M. peltata, M. extensa, P. serratum, P. acerifolium, R. ellipticus, S. multiflora, S. cochinchinensis, V. maderaspatana*, and *W. arborea* are reported for the first time here to inhibit *C. elegans*. However, three plants, namely *A. scholaris* (Singh and Sharma, 2013), *B. racemosa* (Kumar et al., 2011), and *B. monosperma* (Prashanth et al., 2001; Sant et al., 2014), were reported earlier for anthelmintic activity, but did not show significant inhibition of *C. elegans* in the present study. This may be due to differences in test organism, test conditions or concentrations used. Concerning antiviral studies, *A. scholaris* (Antony et al., 2014) and *B. monosperma* (Yadava and Tiwari, 2005), were previously reported, while plants such as *A. bunius, C. graveolens, C. patulus, C. oppositifolia, H. gaitii, M. extensa, P. serratum, R. ellipticus*, and *V. maderaspatana* showed the highest activity and could be suitable for finding antiviral compounds.

Zhu et al. (2011) published a review on nature-derived drugs, where they analyzed “the ranking of drug-productive plant families based on the ratio of the approved drugs to reported bioactive natural products (including leads of the approved and clinical trials drugs)” and concluded that the top-ranked families that produce high numbers of approved drugs among the plant-derived medicines were Fabaceae, Ephedraceae, Papaveraceae, Asteraceae, Solanaceae, Rubiaceae, and Apocynaceae. Girach and his “co-workers” findings (1999) show that eight families appear in the top 15 on the list of high-use families of Similipal viz. Euphorbiaceae, Caesalpiniaceae, Asteraceae, Lamiaceae, Malvaceae, Mimosaceae, Rubiaceae, and Solanaceae. Most of these families are also confirmed active by bioassay in the present study. Our screening results suggest that the top families with bioactivity for infections are Euphorbiaceae, Leguminosae, Apocyanaceae, Phyllanthaceae, Fabaceae, Anacardiaceae, Lamiaceae, and Lecythidaceae.

Although there are a lot of papers published on medicinal plants from India, they mostly neglect the rich traditional knowledge of the tribal peoples. To the best of our knowledge, this paper is the first systematic bioactivity study of medicinal plants from the SBR based on traditional knowledge of indigenous tribes. However, many previous studies have not determined systematically whether such traditionally used species are more likely to yield useful bioactive compounds. It therefore remains controversial whether traditionally used plants form a good starting point for drug discovery (Fabricant and Farnsworth, 2001; Soejarto et al., 2005) and prospection guided by ethnobotany has fluctuated (Cox and Balick, 1994; Firn, 2003; Newman and Cragg, 2016). One view is that “traditionally used medicinal plants are not necessarily efficacious and there are no robust methods for distinguishing those which are most likely to be bioactive when selecting species for further testing” quoted by Saslis–Lagoudakis et al. (2012). Saslis–Lagoudakis et al. (2012), published a review entitled “phylogenies reveal the predictive power of traditional medicine in bioprospecting,” and conclude that “phylogenetic cross-cultural comparisons can focus screening efforts on a subset of traditionally used plants that are richer in bioactive compounds, and could revitalize the use of traditional knowledge in bioprospecting”. Their study provides unique large-scale evidence that plant bioactivity underlies traditional medicine. Our results agree with this view.

## Conclusion

The present study shows the importance of indigenous knowledge of the tribes of the SBR for selecting plants with potential for treating infectious diseases. This knowledge obtained from the tribals and combined with taxonomy, toxicity, and different biological activities demonstrates the value of healing practices and the use of plants by the tribes of the SBR and suggests that bioprospecting of medicinal plants with the help of traditional knowledge of indigenous peoples is useful for the discovery of new drug candidates. However, further scientific studies on isolating the active compounds are essential to discover novel candidate drugs.

## Author contributions

Conceived and designed the experiments: SP, ML, and PL. Performed the experiments: SP, LP, ML, and PL. Analyzed the data: SP, PL, and WL. Contributed reagents/materials/analysis tools: SP, PL, JN, and WL. Contributed to the writing of the manuscript: SP and WL.

### Conflict of interest statement

The authors declare that the research was conducted in the absence of any commercial or financial relationships that could be construed as a potential conflict of interest.

## Abbreviations

ATCC: American Type Culture Collection, Manassas, Virginia, USA
CC: Cell controls
CC_50−_50%: Cytostatic/Cytotoxic Concentration
CPE: Cytopathic effect
DMSO: dimethyl sulphoxide
EC_50_: 50% Effective Concentration
EC_90_: 90% Effective Concentration
EV71: Enterovirus 71
FBS: Fetal bovine serum
LB: Luria-Bertani broth
MDR: Multi–drug resistant
MHA: Mueller–Hinton agar
MIC_50_: Minimum inhibitory concentration required to inhibit the growth by 50% of organisms
MTS: (3–[4,5,dimethylthiazol–2–yl]–5–[3–carboxymethoxy–phenyl]–2–[4– sulfophenyl]–2H–tetrazolium, inner salt
NA: Nutrient agar
NGM: Nematode growth medium
OD: Optical density
RCF: Relative Centrifugal Force
SBR: Similipal Biosphere Reserve
SI: Selectivity index
SS: Selectivity Surface
TI: Therapeutic index
VC: Virus controls
YPD: Yeast extract peptone dextrose medium.
