# Supplementary material for: Antimicrobial, Anthelmintic, and Antiviral Activity of Plants Traditionally Used for Treating Infectious Disease in the Similipal Biosphere Reserve, Odisha, India
Source: Front Pharmacol. 2017 Oct 23;8:658. doi: 10.3389/fphar.2017.00658 (PMC5660100; doi:10.3389/fphar.2017.00658)
Supplement: Supplementary file 1 [file DataSheet1.docx]

Supplementary Material

**Antimicrobial, anthelmintic and antiviral activity of plants traditionally used for treating infectious disease in the Similipal Biosphere Reserve, Odisha, India**

Sujogya Kumar Panda^1,2*^, Laxmipriya Padhi^1^, Pieter Leyssen^3^, Maoxuan Liu^2^, Johan Neyts^3^, Walter Luyten^2^

^1^ Department of Zoology, North Orissa University, Baripada, India

^2^ Department of Biology, KU Leuven, Leuven, Belgium

^3^ Department of Microbiology and Immunology, Rega, Leuven, Belgium

*** Correspondence:** Corresponding Author: Sujogya Kumar Panda

[sujogyapanda@gmail.com](mailto:sujogyapanda@gmail.com)

# Supplementary Data (Antiviral activity of selected extracts)

| *Anogeissus latifolia* | Bark | Acetone |
| --- | --- | --- |

| [*Antidesma bunius*](http://www.theplantlist.org/tpl1.1/record/kew-11964) | Bark | Ethanol |
| --- | --- | --- |

| [*Casearia graveolens*](http://www.theplantlist.org/tpl1.1/record/kew-4702141) | Leaf | Aqueous |
| --- | --- | --- |

| *Melastoma malabathricum* | Leaf | Ethanol |
| --- | --- | --- |

| *Rubus ellipticus* | Leaf | Ethanol |
| --- | --- | --- |

| *Ventilago maderaspatana* | Leaf | Aqueous |
| --- | --- | --- |

| [*Bauhinia racemosa*](http://www.theplantlist.org/tpl1.1/record/ild-824) | Leaf | Ethanol |
| --- | --- | --- |

| *Protium serratum* | Fruit | Ethanol |
| --- | --- | --- |

| *Hypericum gaitii* | Leaf | Aqueous |
| --- | --- | --- |

| *Colebrookea oppositifolia* | Leaf | Aqueous |
| --- | --- | --- |

| *Mimosa rubicaulis* | Wholeplant | Aqueous |
| --- | --- | --- |

| *Glochidion velutinum* | Leaf | Acetone |
| --- | --- | --- |

| *Melastoma malabathricum* | Leaf | Acetone |
| --- | --- | --- |

| *Wrightia arborea* | Leaf | Acetone |
| --- | --- | --- |

| *Wrightia arborea* | Leaf | Aqueous |
| --- | --- | --- |

| [*Millettia extensa*](http://www.theplantlist.org/tpl1.1/record/ild-32243) | Leaf | Aqueous |
| --- | --- | --- |

| *Cleistanthus patulus* | Leaf | Aqueous |
| --- | --- | --- |

| *Rubus ellipticus* | Leaf | Aqueous |
| --- | --- | --- |

**Supplementary figure 1:** Dose response curves for selected extracts of the antiviral (red) and cytotoxic (green) activity

Grey panel to right of each dose responses shows calculated parameters:

EC_50_ = 50% Effective Concentration (concentration at which 50% inhibition of virus replication is observed)

EC_90_ = 90% Effective Concentration (concentration at which 90% inhibition of virus replication is observed)

CC_50_ = 50% Cytostatic/Cytotoxic Concentration (concentration at which 50% adverse effect is observed on RD cells in parallel with antiviral assay)

SI = Selectivity Index (CC_50_/EC_50_)

SS = Selectivity Surface: integrated surface delineated by the EC_50_ curve (red), the CC_50_ curve (green) and the 50% horizontal (black)

TI = Therapeutic Index (SS*10logSI)

**
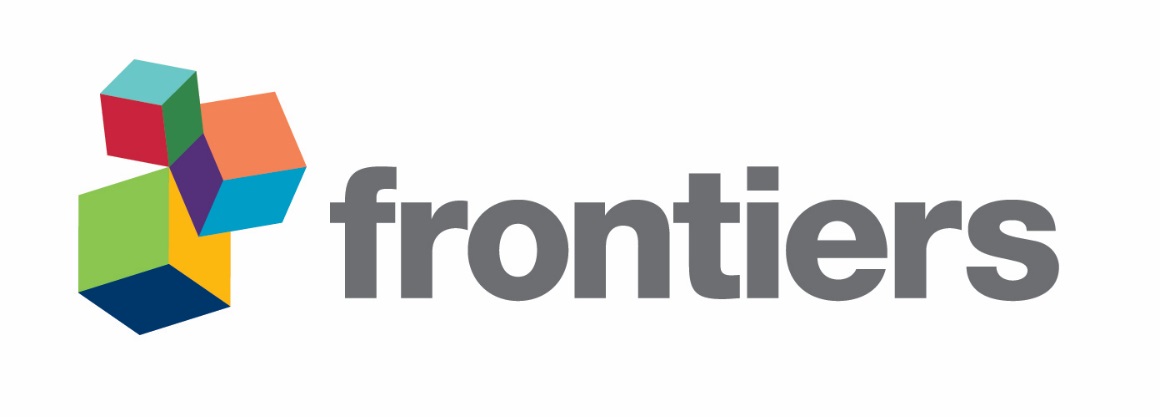
**
